# Eruption-Related Ultraviolet Irradiance Enhancements Associated with Flares

**DOI:** 10.1007/s11207-025-02596-9

**Published:** 2026-01-09

**Authors:** Luke Majury, Marie Dominique, Ryan Milligan, Dana-Camelia Talpeanu, Ingolf Dammasch, David Berghmans

**Affiliations:** 1 Astrophysics Research Centre, School of Mathematics and Physics, Queen’s University Belfast, University Road, BT7 1NN, Northern Ireland Belfast, UK; 2 SIDC, Royal Observatory of Belgium, 3 Avenue Circulaire, 1180 Uccle, Belgium

**Keywords:** Flares, dynamics, Chromosphere, active, Transition region, Heating, in flares, Prominences, active

## Abstract

Large solar flares (GOES M-class or higher) are usually associated with eruptions of material. However, when considering flare irradiance enhancements and dynamics such as chromospheric evaporation, potential contributions from erupted material have historically been neglected. We analyse nine eruptive M- and X-class flares from 2024 to early 2025, quantifying the relative contributions of erupted material to irradiance enhancements during the events. Atmospheric Imaging Assembly (AIA) images from four different channels had ribbon and eruption irradiance contributions separated using a semi-automated masking method. The sample-averaged percentages of excess radiated energy by erupted material over the impulsive phase were $10^{+4}_{-4}\%$, $24^{+14}_{-14}\%$, $21^{+14}_{-10}\%$ and $13^{+6}_{-9}\%$ for the 131 Å, 171 Å, 304 Å and 1600 Å channels, respectively. For three events that were studied in further detail, hard X-ray (HXR) imaging showed little to no signatures of nonthermal heating within the eruptions. Our results suggest that erupted material can be a significant contributor to UV irradiance enhancements during flares, with possible heating mechanisms including nonthermal particle heating, Ohmic heating, or dissipation of MHD waves. Future work may clarify the heating mechanism and evaluate the impact of eruptions on spectral variability, particularly in Sun-as-a-star and stellar flare observations.

## Introduction

Flares are transient bursts of electromagnetic radiation from the solar atmosphere, these events are often associated with eruptions; dynamic ejections of material from either prominences suspended in the corona or directly from the lower layers of the solar atmosphere (Webb and Howard 2012; Raouafi et al. 2016; Shen 2021). Both flares and eruptions occur due to the destabilisation of magnetic flux in the solar corona, often following the emergence of flux from the solar interior. In many cases, instabilities in this flux lead to the eruption of a magnetic flux rope, in the process of which a current sheet is formed (e.g. Heyvaerts, Priest, and Rust 1977; Antiochos, DeVore, and Klimchuk 1999; Moore et al. 2001). As the current sheet thins, the assumption of ideal magnetohydrodynamics (MHD) breaks down (specifically the frozen-in flux condition), resulting in fast magnetic reconnection. This releases magnetic free energy, exceeding $10^{32}\,\mathrm{erg}$ in the largest events. The released energy is partitioned into plasma heating, bulk motions of eruptions, and acceleration of particles to nonthermal energies (Fletcher et al. 2011; Emslie et al. 2012; Aschwanden, Xu, and Jing 2014). Though not all flares have associated coronal mass ejections (CMEs), eruptions that escape the Sun’s low coronal magnetic field, the majority of large flares (GOES M-class or larger) are associated with these successful eruptions, largely due to the magnetic structure of their active regions (Yashiro et al. 2006; Wang and Zhang 2007; Inoue et al. 2013; Zhang et al. 2022; Li et al. 2024).

The radiated energy during major flares can, though infrequently, exceed $10^{32}\,\mathrm{erg}$ in ultraviolet (UV) emission alone (Woods, Kopp, and Chamberlin 2006). This emission originates primarily from chromospheric flare ribbons and coronal loops, and constitutes a large fraction of the total radiated energy (Milligan et al. 2014; Kontar et al. 2017; Warmuth and Mann 2020). Such UV emissions are known to influence the Earth’s ionosphere, ionising neutral particles and driving currents that generate magnetic perturbations, which are observed as a solar flare effect (Sfe; Mitra 1974; Curto 2020). Additionally, the increased ionisation of the ionosphere can interfere with radio communications, as variations in plasma frequency alter the height of the Earth-ionosphere waveguide (Cannon 2013). While the majority of excess UV emissions during flares originate from ribbons and loops, a significant amount of radiation may also originate from eruptions associated with flares. For example, Rubio da Costa et al. (2009) studied an M1.4 class flare with an associated eruption in Ly$\alpha $ images, finding the radiated power from the flare footpoints to be on the order of $10^{26}\,\mathrm{erg}\,\mathrm{s}^{-1}$, corresponding to less than $10\%$ of the available nonthermal power in accelerated electrons. While the associated eruption had a much lower surface intensity of $1.2\times 10^{6}\,\mathrm{erg}\,\mathrm{cm}^{-2}\,\mathrm{s}^{-1}$ than the footpoints at $6.7\times 10^{7}\,\mathrm{erg}\,\mathrm{cm}^{-2}\,\mathrm{s}^{-1}$, the area of erupted material was much larger, suggesting that it may have contributed significantly to the total flare excess in Ly$\alpha $. Contributions of erupted material to the overall Ly$\alpha $ flare excess have also been suggested for flares studied by Milligan (2021) and Wauters et al. (2022). Mierla et al. (2022) reported a bright prominence eruption in He ii 304 Å emission, with the authors attributing its brightness to collisional excitation processes within the prominence. Further analysis by Hayes et al. (2024) of the same event using X-ray observations from the Spectrometer Telescope for Imaging X-rays on Solar Orbiter (SolO/STIX; Krucker et al. 2020) revealed the prominence eruption to be broadly cospatial with soft X-rays (SXRs) of $4-10\,\mathrm{keV}$, with a smaller region of hard X-ray (HXR) emission being seen in the prominence at lower altitudes. From this, the authors inferred heating of the plasma by nonthermal electrons travelling upward from the site of their acceleration. Similar observations of HXR sources associated with eruptions have been reported by Kane et al. (1992), Hudson et al. (2001), Krucker, White, and Lin (2007), Glesener et al. (2013), and Lastufka et al. (2019). Understanding the heating mechanisms (e.g. nonthermal particle heating) driving eruption emission may allow the results of models to be better constrained, with insights into the partitioning of magnetic energy release into erupted and chromospheric plasma being provided (Janvier, Aulanier, and Démoulin 2015; Dahlin et al. 2025).

Contributions of erupting material to the overall flare excess have also been discussed in the context of spectral observations, being suggested as a potential cause of observed blue asymmetries and Doppler shifts, as opposed to chromospheric evaporation (e.g. Batchelor and Hindsley 1991; Ding et al. 2003; Majury et al. 2025; Majury and Milligan 2025). Observations of spectral variability in spatially integrated observations of stellar flares have previously been attributed to both chromospheric evaporation and eruptions (Gunn et al. 1994; Berdyugina, Ilyin, and Tuominen 1999; Wang et al. 2024; Argiroffi et al. 2019). However, recent work by De Wilde et al. (2025) synthesised Sun-as-a-star spectra from imaging spectroscopy observations using the Numerical Empirical Sun-as-a-Star Integrator code (NESSI; Pietrow and Pastor Yabar 2024), finding that spectral variations due to gravitationally-bound upflows could be misinterpreted as signatures of coronal mass ejections (CMEs) in spatially integrated data. Understanding the irradiance contributions of eruptions during flares may help guide future interpretations of spectral variability in spatially integrated flare observations.

In this work, we aim to determine the typical contribution of flare-associated eruptions to the total excess radiated energy. Section 2 provides an overview of the flare identification process, the instruments used, and the methods applied to separate the contributions of flare and eruption emissions to total irradiance enhancement. Section 3 provides results of this analysis, including case studies of specific events and statistical information for a larger sample. Section 4 provides a discussion of, and conclusions from, these results and the broader implications of this work for future research.

## Observations and Analysis

### Flare Identification

Flares were initially chosen for study based on the availability of observations from the Ly$\alpha $ High Resolution Imager telescope of the Extreme Ultraviolet Imager on Solar Orbiter (SolO/EUI HRI_Ly*α*_; Müller et al. 2020; Rochus et al. 2020). This instrument was selected due to previous observations of bright eruptions being made in Ly$\alpha $ (Rubio da Costa et al. 2009; Wauters et al. 2022). This resulted in the identification of two flares, an M2.0 event on 2 March 2022 and an M2.3 event on 7 December 2024. Of these two events, only the M2.0 was eruptive. Thus, to provide more robust statistics, eruptive events that were observed by the Ly$\alpha $ channel of the Large Yield Radiometer on the Project for Onboard Autonomy 2 (PROBA-2/LYRA; Hochedez et al. 2006; Dominique et al. 2013) were sought out. A list of M- and X-class events captured by the backup unit of the instrument in campaigns during 2024 and early 2025 was compiled. Each event was compared to a list of eruptions identified by the Eruption Patrol software module (Hurlburt 2015) from the Heliophysics Event Knowledgebase (HEK; Hurlburt et al. 2012). A subset of flares that had an eruption that was between $50^{\prime \prime}$ and $200^{\prime \prime}$ from the location of the flare and that occurred during the Geostationary Operational Environmental Satellite (GOES) flare period was then identified. Each of 82 events in this subset was then visually inspected in 304 Å images from the Atmospheric Imaging Assembly on the Solar Dynamics Observatory (SDO/AIA; Pesnell, Thompson, and Chamberlin 2012; Lemen et al. 2012) using the JHelioviewer visualisation software package (Müller et al. 2017). For each event, the following criteria were applied:

- Flare had an associated eruption that appeared brighter than the quiet Sun.
- Eruption must have originated from the flare active region.
- Flare ribbons and eruption must have had minimal spatial overlap.

Applying these criteria resulted in the generation of a final list of nine events (including the event observed by HRI_Ly*α*_) for which the radiative properties of the flare ribbons and associated eruption emission were analysed in detail. Details of these events are listed in Table 1.

**Table 1:** GOES start, peak, and end times (UT) along with coordinates and active region numbers for the sample of nine eruptive events.

Class	Date	Start Time	Peak Time	End Time	Stonyhurst Coordinate (°)	NOAA AR Number
M1.1	2024-03-23	06:47	06:55	06:59	S13E04	13615
M1.1	2025-01-07	22:35	23:05	23:42	S20W88	13939
M1.9	2024-12-21	00:33	00:38	00:42	S15E61	13932
M2.0	2022-03-02	17:31	17:39	17:47	N15E29	12958
M2.7	2025-01-24	20:48	21:04	21:17	S06W67	13961
M3.8	2024-12-19	15:27	15:34	15:39	S14E81	13928
X1.0	2024-05-12	16:11	16:26	16:38	S18W72	13664
X2.0	2024-10-31	21:12	21:20	21:27	N15E28	13878
X9.0	2024-10-03	12:08	12:18	12:27	S15W03	13842

### Separation of Eruption and Ribbon Contributions in UV Images

#### UV Imagers

Data from three UV imaging instruments were included in this analysis. Firstly, and primarily, data from SDO/AIA were used. AIA provides high-cadence ($12\,\mathrm{s}$) imaging of the entire solar disk in 10 different wavelength bands, covering a wide range of temperatures and hence probing various regions of the solar atmosphere. These specifications enable AIA to provide near-complete coverage of solar flares at a cadence suitable for studying their dynamics. In this work, we employed AIA observations of nine flares with associated bright eruptions, using a mask to spatially separate ribbon and eruption contributions to total flare excess radiated energy.

For a single eruptive event, an M2.0 flare on 2 March 2022, further imaging from the high-resolution Ly$\alpha $ and Extreme Ultraviolet (EUV) telescopes of SolO/EUI was used to supplement AIA data. HRI_Ly*α*_ and HRI_EUV_ provide images at a resolution of $\sim 3^{\prime \prime}$ and $\sim 1^{\prime \prime}$, respectively. The absolute resolution (km) of each instrument varies with the orbital radius of SolO. Furthermore, HRI_Ly*α*_ experiences temperature-driven degradation to its resolution, particularly near perihelion (Berghmans et al. 2023). Fortunately, the eruptive event that was observed occurred while SolO was near aphelion.

Additionally, for an occulted M1.1 flare on 7 January 2025, images from the Extreme Ultraviolet Imager of the Sun-Earth Connection Coronal and Heliospheric Investigation on the Solar-Terrestrial Relations Observatory (STEREO/SECCHI EUVI; Kaiser et al. 2008; Howard et al. 2008; Wuelser et al. 2004) were used to analyse emissions from the flare ribbons, which were visible from STEREO-A. The EUVI instrument operates with a modest cadence, as fast as $2.5\,\mathrm{minutes}$, in four wavelength channels: 171 Å, 195 Å, 284 Å, and 304 Å.

#### Ribbon and Eruption Masking

**Figure 1:**
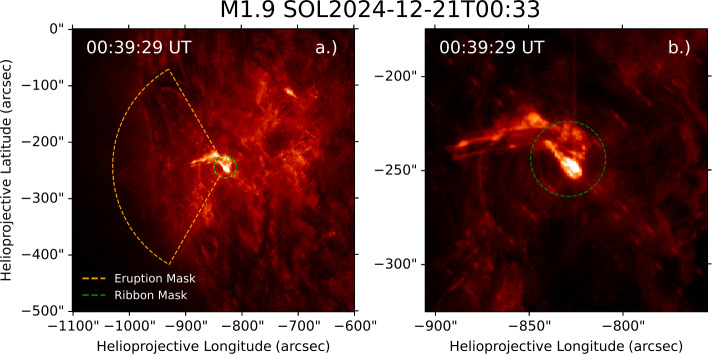
AIA 304 Å images of an M1.9 flare on 21 December 2024 with eruption and ribbon masks overlaid. (a) Shows a global view of the event. (b) A close-up of the flare and eruption.

For each event in the sample, masks were constructed around the AIA 304 Å frame with the highest count rate ($\mathrm{DN}\,\mathrm{s}^{-1}$) during the flare. Ribbon masks were defined as a circle sharing a centre with the largest $20\%$ intensity contour, with a radius covering the entirety of the ribbons determined by visual inspection. The eruption masks were defined as a circular sector, sharing a common centre with the corresponding ribbon mask, extending from the edge of the ribbon mask to a manually chosen upper radius. The upper and lower angles of this sector were similarly manually defined. The manual choices in mask selection were made conservatively. Thus, regions with overlapping eruption and ribbon emission were absorbed into the ribbon mask. An example of the eruption and ribbon masks chosen for an M1.9 flare in the sample is shown in Figure 1, where panel a shows a global view of the event while panel b shows a close-up of the eruption and ribbons.

For each flare, AIA images in four channels (1600 Å, 304 Å, 171 Å, and 131 Å) from 30 minutes before the GOES start time to 30 minutes after the GOES end time were analysed. This allowed the relative contribution of eruptions to emissions at different formation temperatures to be ascertained. The ribbon and eruption masks were applied to each image in each channel, with the count rates of pixels within each mask being summed. This provided total count rates of both the ribbons and the eruption for each frame. The count rate values for each channel were then radiometrically calibrated using the AIA response function provided by the get_aia_response routine in SolarSoft (Freeland and Handy 1998), providing the radiated energy rates for each image in $\mathrm{erg}\,\mathrm{s}^{-1}$. Total (ribbon plus eruption) radiated energies during the flare impulsive phases (GOES start to GOES peak time) were then calculated for each wavelength, with radiated energies over the full flare durations (GOES start to GOES end time) additionally being calculated for the 304 Å and 1600 Å channels.

For the eruptive flare observed by HRI_Ly*α*_ and HRI_EUV_, the same masking technique as described for AIA was applied, with the equivalent mask being reprojected to SolO’s point of view during the event. Unfortunately, the calibrated response functions for HRI are not yet available, so calculation of radiated energies in absolute units was not possible. As the event observed by EUVI was occulted from the perspective of SDO, a mask was manually reconstructed using the same approach described for AIA above, rather than reprojecting. As with HRI, radiometric calibration was not performed for EUVI, with only the fraction of excess counts in the ribbon and eruption masks being determined. For both EUI and EUVI, light travel time corrections were applied, synchronising their observations with Earth-based instruments.

### X-ray Imaging

For three events with available data, HXR observations from the Spectrometer/Telescope for Imaging X-rays (STIX) or the Hard X-ray Imager on the Advanced Space-based Solar Observatory (ASO-S/HXI; Gan et al. 2019; Zhang et al. 2019) were used to generate HXR images. This allowed any spatial overlap between HXRs, which infer heating by nonthermal particles, and erupted material to be identified. STIX provides spectrally-resolved imaging of solar X-rays with energies between 4 and $150\,\mathrm{keV}$. The instrument employs a Fourier imaging technique, with incident X-ray flux being spatially modulated in a unique Moiré pattern for each of 30 subcollimator-detector pairs. The amplitude and phase of each measured pattern provide Fourier visibilities from which images can be reconstructed. HXI provides similar spectrally-resolved imaging of solar X-rays from Earth orbit. HXI also employs a spatially modulated Fourier imaging technique, with 91 detector-subcollimator pairs. The maximum entropy method (MEM_GE; Massa et al. 2020) was applied to STIX data to generate images, with the Clean algorithm (Högbom 1974) being applied to HXI data.

### UV Photometry

In this study, photometric data from PROBA-2/LYRA and the B-channel of the Extreme Ultraviolet Sensor of the Extreme Ultraviolet and X-ray Irradiance Sensors on GOES (GOES/EXIS EUVS-B; Eparvier et al. 2009) were used to determine whether observed irradiance contributions from bright eruptions can be seen in disk-integrated data. This was examined by comparing spatially resolved AIA 304 Å data and spatially integrated observations from EUVS-B and LYRA.

EUVS-B provides moderate-cadence ($30\,\mathrm{s}$) observations of solar irradiance between 1180 Å – 1270 Å, dominated by Ly$\alpha $ and Si iii emission. Its predecessor instruments on the GOES 13, 14, and 15 satellites have been widely employed to study Ly$\alpha $ flare variability (Milligan et al. 2020; Lu et al. 2021; Milligan 2021; Greatorex, Milligan, and Chamberlin 2023). PROBA-2/LYRA provides very high-cadence (nominally $50\,\mathrm{ms}$) observations of solar irradiance during flares in four EUV channels: Ly$\alpha $, Herzberg, aluminium, and zirconium. The instrument carries three nearly identical units, with the backup unit (unit 1) of the instrument currently maintaining a relatively high signal in its Ly$\alpha $ channel. Past flare studies using the instrument are presented in Kretzschmar, Dominique, and Dammasch (2013) and Wauters et al. (2022).

## Results

### The M2.0 Flare on 2 March 2022

**Figure 2:**
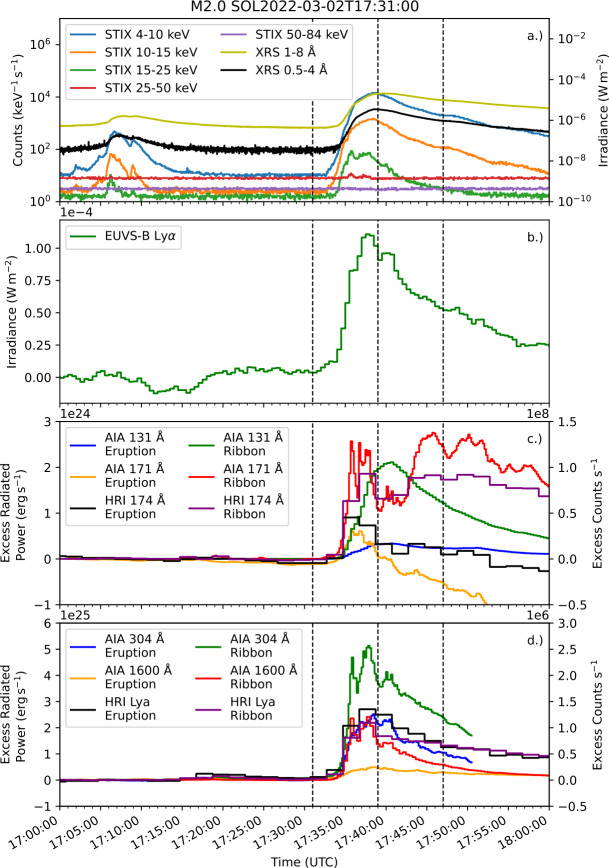
X-ray and UV emission during an M2.0 flare on 2 March 2022. (a) shows lightcurves from STIX and XRS. Excess Ly$\alpha $ emission measured by EUVS-B is displayed in (b). (c) and (d) show spatially-separated flare excesses from the flare eruption and ribbons from four AIA channels (radiated power) and the two channels of HRI (count rate). Black dashed lines indicate GOES start, peak, and end times from left to right.

HRI_Lya_ has observed a small number of large flares to date, including an M2.0 flare on 2 March 2022. The event was observed from a similar perspective to Earth-based instruments, with SolO close to aphelion. Lightcurves of the event are presented in Figure 2, with X-ray observations from STIX and the X-ray Sensor (XRS; Chamberlin et al. 2009) on GOES presented in panel a, and Ly$\alpha $ observations from EUVS-B plotted in panel b. Panel c shows excess radiated power from the eruption and ribbons for the 131 Å and 171 Å channels of AIA, as well as the excess count rates for HRI_EUV_. Similar values for AIA 304 Å, and 1600 Å, along with HRI_Lya_ are presented in panel d. From GOES start to peak time (i.e. the flare impulsive phase), the percentages of the total flare excess energy radiated by the eruption were $15\%$, $41\%$, $34\%$, $19\%$ for the 131 Å, 171 Å, 304 Å, and 1600 Å channels of AIA, respectively. The fractions of total excess counts in the eruption measured by HRI_Lya_ and HRI_EUV_ were $18\%$ and $55\%$, respectively. The timing of peak eruption emission was within $36\,\mathrm{s}$ of the peak in the ribbons for AIA 304 Å. This close temporal overlap resulted in the ribbon and eruption peaks being indistinguishable in photometric Ly$\alpha $ observations, making inference of an eruption impossible from these observations alone.

**Figure 3:**
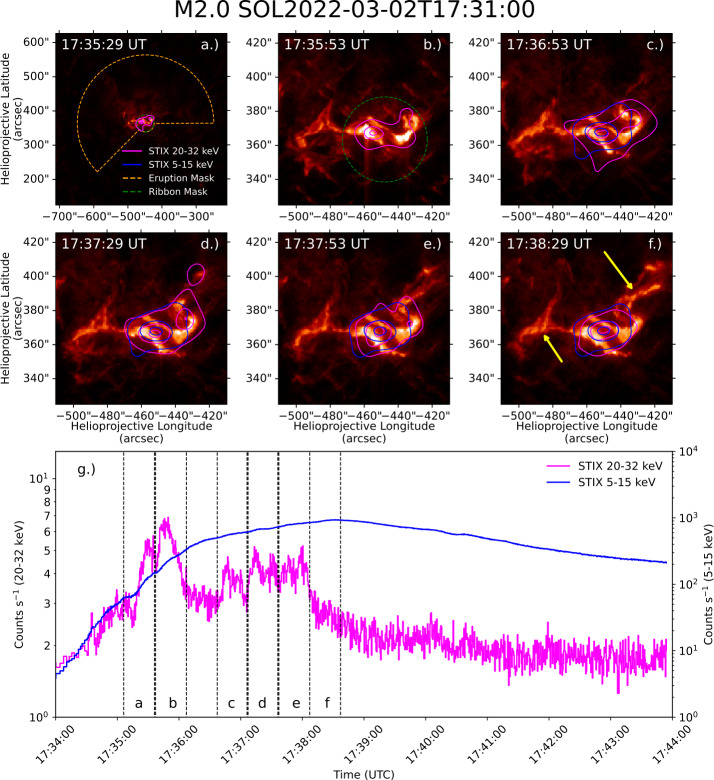
AIA 304 Å images of the M2.0 flare on 2 March 2022 with HXR image contours at 30%, 50%, 70%, and 90% overlaid, shown in panels a through f. Also shown in panel a is the mask used to spatially partition the eruption and ribbon contributions to overall flare irradiance enhancement. (b) Shows a closer view of the ribbon mask. In panel f, yellow arrows indicate the locations of erupted material. (g) Shows X-ray lightcurves from STIX for emission from $20-32\,\mathrm{keV}$ (pink) and $5-15\,\mathrm{keV}$ (blue), with the time intervals over which each HXR image in panels a.) – f.) was generated indicated by vertical dashed lines.

Figure 3 shows the evolution of the M2.0 flare and associated eruption in AIA 304 Å images, with MEM_GE image contours for SXR ($5-15\,\mathrm{keV}$) and HXR ($20-32\,\mathrm{keV}$) emission overlaid. Each panel represents a different time interval during the event, with panels a and b showing X-ray emission spatially coincident with ribbons seen in AIA 304 Å. In panels c and d, material can be seen erupting near the northmost ribbon, with a HXR source extending in the direction of this eruption. Panel d shows a distinct HXR source overlapping with this eruption, indicating nonthermal particle heating within the eruption. However, panels e and f show the ribbons at peak brightness, with a lack of HXR or SXR emission sources in the bright erupted material. Notably, SXR emission did not overlap with the eruption, indicating its temperature to be relatively cooler than the flare loops. Panel g shows nonthermal ($20-32\,\mathrm{keV}$) and thermal ($5-15\,\mathrm{keV}$) X-ray lightcurves from STIX, with the integration times used to reconstruct each set of images indicated by dashed black lines.

### The X9.0 Flare on 3 October 2024

**Figure 4:**
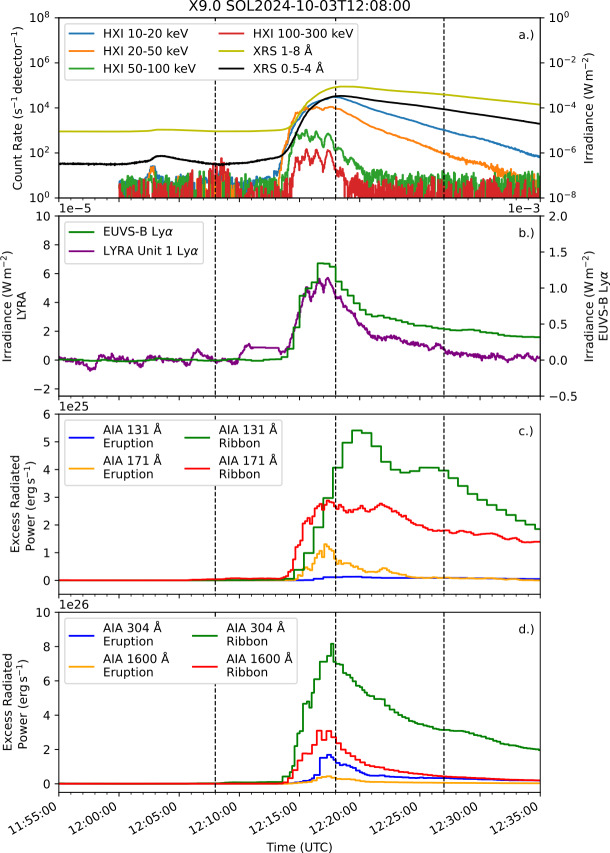
X-ray and UV emission during the X9.0 flare on 3 October 2024. (a) Shows lightcurves from HXI and XRS. Excess Ly$\alpha $ emission measured by EUVS-B and LYRA is displayed in panel b. (c) and (d) Show spatially-separated excess radiated power from the flare eruption and ribbons from four AIA channels. Black dashed lines indicate GOES start, peak, and end times from left to right.

To date, the largest flare of Solar Cycle 25 was the X9.0 event on 3 October 2024. Lightcurves for the event in X-ray and UV wavelengths are presented in Figure 4, with panel a showing X-ray observations from XRS and HXI, and panel b displaying Ly$\alpha $ observations from LYRA (unit 1) and EUVS-B. Panel c displays the eruption and ribbon excess radiated power for AIA 131 Å and 171 Å. The equivalent radiated powers for AIA 304 Å and 1600 Å are shown in panel d. During the impulsive phase, the fractions of total excess energy radiated by the eruption were $7\%$, $22\%$, $12\%$, and $10\%$ for the 131 Å, 171 Å, 304 Å, and 1600 Å channels, respectively. The relatively large fraction of energy radiated in the eruption for the 171 Å channel is likely driven in part by pixel bleed of ribbon emission into the eruption mask, which is mitigated for the 304 Å and 131 Å channels through short exposures. For this reason the event is excluded from statistics of 171 Å emission in Section 3.4. The timing of peak enhancement, as in the M2.0 event, is similar for ribbons and eruption, with the eruption peaking $15\,\mathrm{s}$ before the ribbons in 304 Å emission. This closeness in time of their emission again makes the signatures of eruption emission unclear in Ly$\alpha $ photometric observations. The fast cadence of LYRA (integrated here to $3\,\mathrm{s}$) shows three distinct peaks in the impulsive phase, corresponding to three bursts of HXR emission seen in HXI $100-300\,\mathrm{keV}$ emission, indicating a likely nonthermal origin to this Ly$\alpha $ flare emission. These bursts illustrate short-period quasi-periodic pulsations in the event, which have been studied in further detail using HXI data in Li (2025). Though a halo CME occurs during the event, as identified in the CACTUS archive, little to no coronal dimming is seen in the ribbon or eruption lightcurves in 171 Å emission.

**Figure 5:**
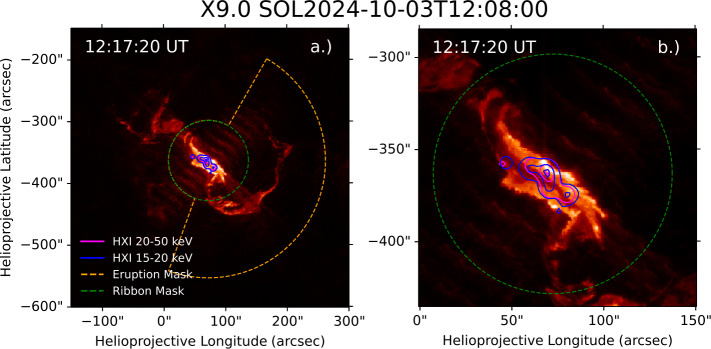
AIA 304 Å images of the X9.0 flare on 3 October 2024 with HXR image contours at 10%, 50%, and 90%, and the mask used to spatially partition the eruption and ribbon contributions to overall flare irradiance enhancement overlaid. HXR images were generated for energies of $20-50\,\mathrm{keV}$ and $15-20\,\mathrm{keV}$ over a time interval of $30\,\mathrm{s}$ (12:17:00 – 12:17:30 UT). (a) Shows a global view of the event. A close-up of the flare is shown in (b).

AIA 304 Å images of the flare are displayed in Figure 5, with CLEAN images generated using HXI observations of X-rays between $15-20\,\mathrm{keV}$ and $20-50\,\mathrm{keV}$ overlaid, and the respective ribbon and eruption masks overplotted. The bright eruption was spatially distinct from the primary HXR sources, suggesting it was likely not strongly heated by nonthermal particles.

### The M1.1 Flare on 7 January 2025

**Figure 6:**
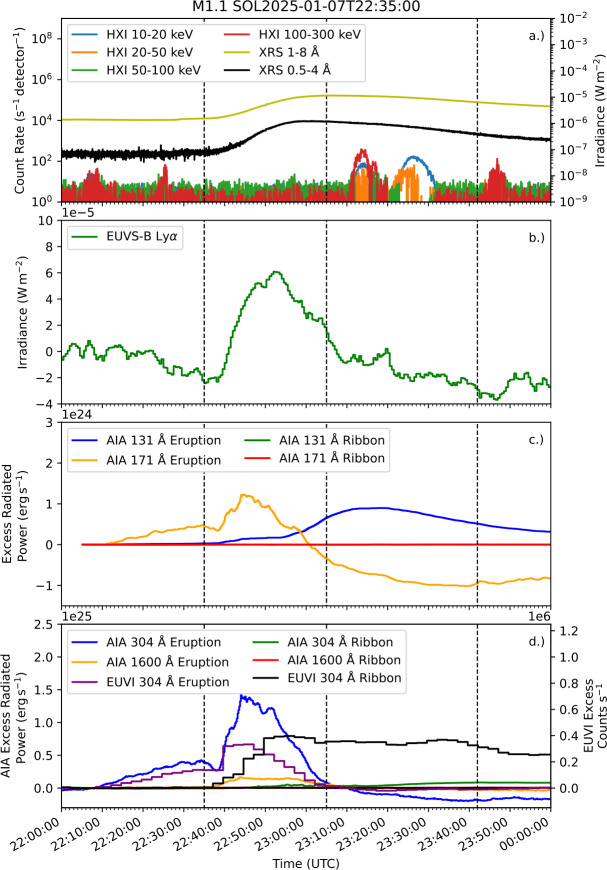
X-ray and UV emission during an M1.1 flare on 7 January 2025. (a) Shows X-ray lightcurves from HXI and XRS. Excess Ly$\alpha $ emission measured by EUVS-B is displayed in (b). (c) and (d) Show spatially-separated excess emission from the flare eruption and ribbons from four AIA channels (radiated power) and the 304 Å channel of EUVI (count rate). Black dashed lines indicate GOES start, peak, and end times from left to right.

The M1.1 flare on 7 January 2025 is the sole behind-the-limb flare of our sample. Figure 6 shows lightcurves of the event, with X-ray observations from XRS and HXI shown in panel a. Ly$\alpha $ observations from EUVS-B are shown in panel b. Panel c shows excess radiated power by the eruption and a quiet-Sun region in AIA 131 Å and 171 Å emission. Similarly, panel d shows excess radiated power in AIA 304 Å and 1600 Å emission, along with excess count rates in 304 Å emission measured by EUVI. Over the impulsive phase, the percentage of excess energy radiated by the eruption was close to $100\%$ in each AIA channel, due to the occultation of the ribbons. For EUVI, which observed both eruption and ribbons, $41\%$ of the excess counts were radiated by the eruption over the impulsive phase in 304 Å emission. Assuming a similar fraction for AIA implies an excess radiated energy by the ribbons of $\sim 2.5\times 10^{28}\,\mathrm{erg}$. HXI lightcurves show no HXR emission associated with the eruption, suggesting that the eruption brightening was not driven by local nonthermal heating. Coronal dimming was seen in 171 Å emission, similarly to the M2.0 event in Section 3.1. A CME associated with the event is listed in the Computer Aided CME Tracking (CACTus) archive, indicating that the eruption was successful. EUVI observations show peak ribbon emission at 22:45:53 UT, 10 minutes after the peak brightness of the eruption. This behaviour in timing somewhat resembles that seen in flare hot X-ray onsets (e.g. Hudson et al. 2021; Battaglia et al. 2023). However, the eruption emission appears relatively cooler than these onsets, which have temperatures $>10^{7}\,\mathrm{K}$, appearing brightest in He ii 304 Å emission that forms at $\mathrm{T}=10^{4.9}\,\mathrm{K}$.

**Figure 7:**
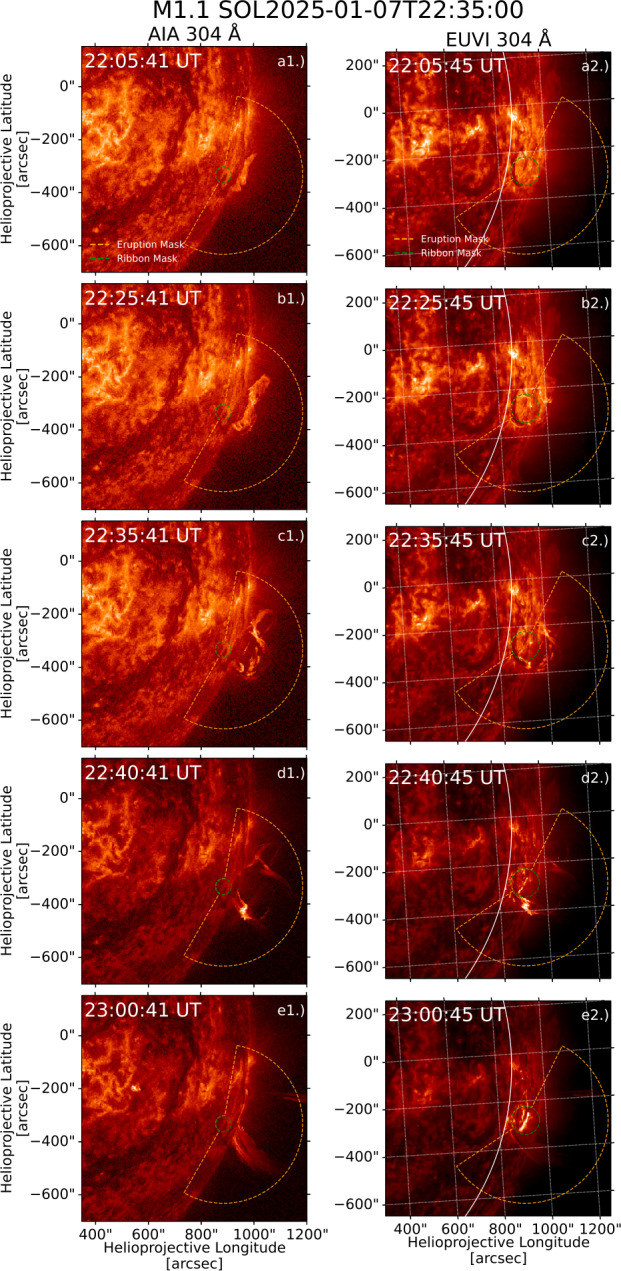
AIA (left column) and EUVI (right column) 304 Å images of an M1.1 flare on 7 January 2025, with the eruption and ribbon masks applied for each set of images overplotted in each panel. The solar limb as seen from AIA is plotted as a white line on each EUVI image.

Images of the flare in 304 Å emission from AIA and EUVI are plotted in the left and right columns of Figure 7, respectively, with eruption and ribbon masks for each instrument overplotted. Panels a1 – b1 and a2,– b2 show an erupting filament, with the structure appearing to unravel in panels c1 and c2. The structure shows clear brightening in panels d1 and d2, with flare ribbons visible in the EUVI image. Panels e1 and e2 show the erupted material post-brightening, with the ribbons appearing strongest in e2.

### Statistics from Nine M- and X-Class Events

The masking technique to separate flare ribbon and eruption irradiance contributions was applied to a further six events, providing small-sample insights into the typical contributions of eruptions to irradiance enhancements during M- and X-class flares. Radiated energies over the impulsive phase by ribbons and eruptions for 131 Å, 171 Å, 304 Å, and 1600 Å emission measured by AIA, along with the percentages of energy radiated by the eruptions, are presented in Table 2. Across the sample, the average percentage of energy radiated by the eruption was greatest for 171 Å emission at $24^{+14}_{-14}\%$, with averages of $10^{+4}_{-4}\%$, $21^{+14}_{-10}\%$, and $13^{+6}_{-9}\%$ for the 131 Å, 304 Å and, 1600 Å channels, respectively. The plus and minus errors in these percentages represent the difference between the sample mean and the sample upper and lower quartile values, respectively. These percentages correspond to absolute radiated energies by eruptions of $2.43\times 10^{26}\,\mathrm{erg}$, $9.43\times 10^{25}\,\mathrm{erg}$, $5.29\times 10^{27}\,\mathrm{erg}$, and $1.36\times 10^{27}\,\mathrm{erg}$ for the respective channels.

**Table 2:** Impulsive phase (GOES start to peak time) radiated energies (erg) for eruptions and ribbons with percentage of total (eruption and ribbon) energy radiated by the eruption in 131 Å, 171 Å, 304 Å, and 1600 Å emission for nine M- and X-class flares. Average radiated energies and percentages of energy radiated by eruption for the events (*excluding the M1.1 on 7 January 2025 for all channels, and the X9.0 flare on 3 October 2024 for the 171 Å channel) are also listed. Times are in UT.

Class	Start Time	131 Å	171 Å	304 Å	1600 Å
Eruption Energy (erg)					
M1.1	2024-03-23 06:47	2.41 × 10^25^	2.41 × 10^26^	3.92 × 10^27^	8.45 × 10^26^
M1.1*	2025-01-07 22:35	3.44 × 10^26^	2.83 × 10^27^	1.73 × 10^28^	2.52 × 10^27^
M1.9	2024-12-21 00:33	1.49 × 10^24^	1.26 × 10^25^	2.05 × 10^26^	1.80 × 10^25^
M2.0	2022-03-02 17:31	5.30 × 10^25^	3.32 × 10^26^	5.28 × 10^27^	1.06 × 10^27^
M2.7	2025-01-24 20:48	3.09 × 10^25^	2.42 × 10^26^	1.91 × 10^27^	4.97 × 10^26^
M3.8	2024-12-19 15:27	8.93 × 10^24^	2.35 × 10^25^	4.23 × 10^26^	2.83 × 10^25^
X1.0	2024-05-12 16:11	2.40 × 10^26^	4.47 × 10^26^	5.86 × 10^27^	1.84 × 10^27^
X2.0	2024-10-31 21:12	1.64 × 10^26^	4.04 × 10^26^	9.31 × 10^27^	1.54 × 10^27^
X9.0	2024-10-03 12:08	2.32 × 10^26^	1.45 × 10^27^	1.54 × 10^28^	5.06 × 10^27^
Ribbon Energy (erg)					
M1.1	2024-03-23 06:47	1.19 × 10^26^	4.49 × 10^26^	6.63 × 10^27^	1.30 × 10^27^
M1.1*	2025-01-07 22:35	2.36 × 10^24^	1.11 × 10^25^	5.26 × 10^26^	6.44 × 10^25^
M1.9	2024-12-21 00:33	2.27 × 10^25^	1.05 × 10^26^	1.48 × 10^27^	9.23 × 10^26^
M2.0	2022-03-02 17:31	3.07 × 10^26^	4.85 × 10^26^	1.02 × 10^28^	4.48 × 10^27^
M2.7	2025-01-24 20:48	8.27 × 10^26^	7.67 × 10^26^	1.28 × 10^28^	4.97 × 10^27^
M3.8	2024-12-19 15:27	5.15 × 10^25^	2.45 × 10^26^	4.92 × 10^27^	1.95 × 10^27^
X1.0	2024-05-12 16:11	1.54 × 10^27^	6.72 × 10^26^	8.46 × 10^27^	7.29 × 10^27^
X2.0	2024-10-31 21:12	2.87 × 10^27^	5.20 × 10^27^	9.36 × 10^28^	3.49 × 10^28^
X9.0	2024-10-03 12:08	3.05 × 10^27^	5.13 × 10^27^	1.18 × 10^29^	4.79 × 10^28^
Percentage of Total Radiated Energy by Eruption (%)					
M1.1	2024-03-23 06:47	17	35	37	39
M1.1*	2025-01-07 22:35	99	100	97	98
M1.9	2024-12-21 00:33	6	11	12	2
M2.0	2022-03-02 17:31	15	41	34	19
M2.7	2025-01-24 20:48	4	24	13	9
M3.8	2024-12-19 15:27	15	9	8	1
X1.0	2024-05-12 16:11	13	40	41	20
X2.0	2024-10-31 21:12	5	7	9	4
X9.0	2024-10-03 12:08	7	22*	12	10
Averages					
Eruption Energy (erg)	–	9.43 × 10^25^	2.43 × 10^26^	5.29 × 10^27^	1.36 × 10^27^
Ribbon Energy (erg)	–	1.10 × 10^27^	1.13 × 10^27^	3.20 × 10^28^	1.30 × 10^28^
Eruption Percentage (%)	–	$10^{+4}_{-4}$	$24^{+14}_{-14}$	$21^{+14}_{-10}$	$13^{+6}_{-9}$
Fraction IQR (%)	–	9	28	24	16

The greatest radiated energy by an eruption was $1.73\times 10^{28}\,\mathrm{erg}$ in 304 Å emission for the M1.1 flare on 7 January 2025, with the most energy radiated by ribbons being $1.19\times 10^{29}\,\mathrm{erg}$, also in 304 Å emission, during the X9.0 event. The fraction of total energy radiated by an eruption was also greatest during the M1.1 on 7 January 2025, at $100\%$ for 171 Å emissions, due to the event ribbons being occulted. Aside from this event, in AIA data, the largest percentage of total radiated energy by an eruption was $41\%$ for 304 Å emissions during the X1.0 flare. However, HRI_Ly*α*_ measured a larger $55\%$ during the M2.0 event, potentially due to the line large optical depth in the chromosphere, with erupted material perhaps more efficiently radiating away energy in the line due to lower opacity in the corona (Fontenla, Reichmann, and Tandberg-Hanssen 1988).

Gradual phase contributions were not considered for 131 Å and 171 Å emissions due to potential contamination from flare loop emission and coronal dimming. However, Table 3 lists eruption and ribbon radiated energies over the entire GOES flare durations for 304 Å and 1600 Å emissions. The averaged energies for both eruptions and ribbons are expectedly greater than the impulsive phase values presented in Table 2. The percentage of energy radiated by the eruption is also greater over the entire flare duration than the impulsive phase alone for both channels, suggesting that eruption emission is relatively stronger in the gradual phase. However, this result has weak significance due to the small sample size.

**Table 3:** Radiated energies (erg) over GOES flare period for eruptions and ribbons with percentage of total (eruption and ribbon) energy radiated by the eruption in 304 Å and 1600 Å emission for nine M- and X-class flares. Average radiated energies and percentages of energy radiated by the eruption for the events (*excluding M1.1 on 7 January 2025) are also listed. Times are in UT.

Eruption Energy (erg)			
Class	Start Time	304 Å	1600 Å
M1.1	2024-03-23 06:47	1.31 × 10^28^	1.69 × 10^27^
M1.1*	2025-01-07 22:35	1.93 × 10^28^	3.24 × 10^27^
M1.9	2024-12-21 00:33	1.98 × 10^27^	1.78 × 10^26^
M2.0	2022-03-02 17:31	1.28 × 10^28^	2.69 × 10^27^
M2.7	2025-01-24 20:48	3.98 × 10^27^	8.98 × 10^26^
M3.8	2024-12-19 15:27	3.63 × 10^27^	2.72 × 10^26^
X1.0	2024-05-12 16:11	1.49 × 10^28^	3.97 × 10^27^
X2.0	2024-10-31 21:12	2.31 × 10^28^	3.34 × 10^27^
X9.0	2024-10-03 12:08	4.56 × 10^28^	1.20 × 10^28^
Ribbon Energy (erg)			
M1.1	2024-03-23 06:47	1.03 × 10^28^	1.98 × 10^27^
M1.1*	2025-01-07 22:35	1.91 × 10^27^	1.33 × 10^26^
M1.9	2024-12-21 00:33	6.37 × 10^27^	2.15 × 10^27^
M2.0	2022-03-02 17:31	2.53 × 10^28^	9.22 × 10^27^
M2.7	2025-01-24 20:48	2.26 × 10^28^	7.62 × 10^27^
M3.8	2024-12-19 15:27	1.07 × 10^28^	4.11 × 10^27^
X1.0	2024-05-12 16:11	2.25 × 10^28^	1.15 × 10^28^
X2.0	2024-10-31 21:12	1.59 × 10^29^	4.83 × 10^28^
X9.0	2024-10-03 12:08	3.70 × 10^29^	1.01 × 10^29^
Percentage of Total Radiated Energy by Eruption (%)			
M1.1	2024-03-23 06:47	56	46
M1.1*	2025-01-07 22:35	91	96
M1.9	2024-12-21 00:33	24	8
M2.0	2022-03-02 17:31	34	23
M2.7	2025-01-24 20:48	15	11
M3.8	2024-12-19 15:27	25	6
X1.0	2024-05-12 16:11	40	26
X2.0	2024-10-31 21:12	13	6
X9.0	2024-10-03 12:08	11	11
Averages			
Eruption Energy (erg)		1.49 × 10^28^	3.13 × 10^27^
Ribbon Energy (erg)		7.84 × 10^28^	2.32 × 10^28^
Eruption Percentage (%)		$27^{+8}_{-13}$	$17^{+6}_{-10}$
Fraction IQR (%)		21	16

Table 4 shows the timing of peak eruption and ribbon emission for the 304 Å and 1600 Å channels. The other channels are again excluded due to flare loop contributions and coronal dimming. It is seen that the eruption intensity peaks after or at the same time as the ribbons for seven of eight events in the 304 Å emission and for all eight events in 1600 Å, omitting the M1.1 event, which had occulted ribbons. This may suggest that a heating mechanism other than the deposition of nonthermal electron energy drives the enhancement of erupted material. Other possible mechanisms may include conduction of energy from the ribbons or heating through Ohmic dissipation of reconnection-driven currents. The largest time delay between peak ribbon and eruption emission occurred during the X2.0 event, which had its eruption emission peak over 30 minutes after its ribbons. For the M1.1 flare on 7 January 2025, the flare ribbons were not visible from AIA. However, EUVI observations show that the event eruption peaks in brightness around 10 minutes before the ribbons. This indicates that while eruption and ribbon emissions typically peak close together in time, peak eruption emission can occur tens of minutes either before or after the ribbon peak.

**Table 4:** Timing of peak AIA 304 Å and 1600 Å emission from eruption and ribbons for nine M- and X-class flares, with time difference between eruption and ribbon peak. *Timing differences not noted for M1.1 flare as ribbons were occulted.

Class	Date	Start Time	Eruption Peak Time	Ribbon Peak Time	Δ t (s) (Erupt-Rib)
304 Å					
M1.1	2024-03-23	06:47:00	06:57:05	06:54:41	144
M1.1*	2025-01-07	22:35:00	22:44:05	N/A	N/A
M1.9	2024-12-21	00:33:00	00:38:29	00:37:53	36
M2.0	2022-03-02	17:31:00	17:38:29	17:37:53	36
M2.7	2025-01-24	20:48:00	21:04:41	20:56:17	504
M3.8	2024-12-19	15:27:00	15:35:53	15:33:53	120
X1.0	2024-05-12	16:11:00	16:21:53	16:19:29	144
X2.0	2024-10-31	21:12:00	21:49:53	21:16:55	1977
X9.0	2024-10-03	12:08:00	12:17:29	12:17:44	−15
1600 Å					
M1.1	2024-03-23	06:47:00	06:55:02	06:55:02	0
M1.1*	2025-01-07	22:35:00	22:43:50	N/A	N/A
M1.9	2024-12-21	00:33:00	00:38:38	00:37:50	48
M2.0	2022-03-02	17:31:00	17:38:14	17:37:50	24
M2.7	2025-01-24	20:48:00	21:04:38	20:55:50	528
M3.8	2024-12-19	15:27:00	15:35:50	15:33:50	120
X1.0	2024-05-12	16:11:00	16:19:26	16:19:26	0
X2.0	2024-10-31	21:12:00	21:49:02	21:15:50	1992
X9.0	2024-10-03	12:08:00	12:17:26	12:16:38	48

Examining EUVS-B Ly$\alpha $ data for each event reveals an average peak flare percentage enhancement above background of $3.16\%$, with the first data point after the GOES start time considered as a background. Such a value is consistent with typical values for M- and X-class events, suggesting that flare-associated eruptions may not drive significantly larger enhancements (Milligan et al. 2020; Milligan 2021). Few events in the sample showed appreciable Ly$\alpha $ enhancements in LYRA observations, the largest enhancement being during the X9.0 event at $\sim 1\%$, considerably less than the $13\%$ measured by EUVS-B for the same event. This disparity may have been due to contamination of the LYRA bandpass by continuum emission (Greatorex, Milligan, and Dammasch 2024).

### Eruption Detection in Photometric Observations

**Figure 8:**
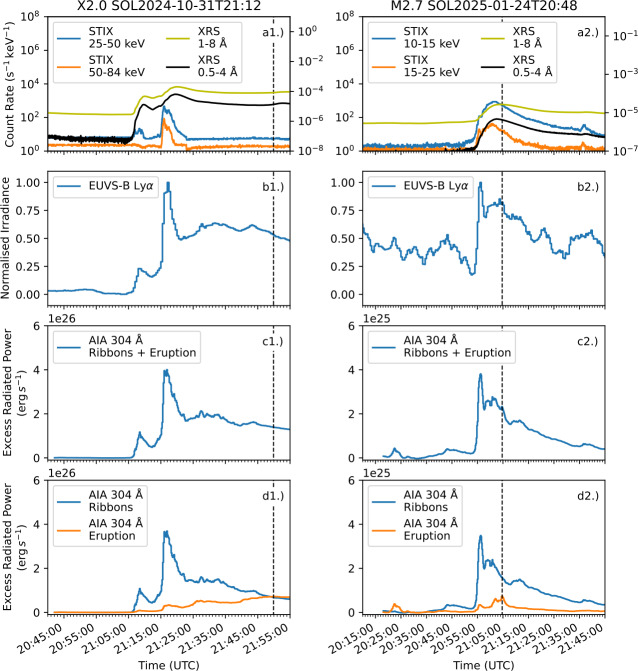
X-ray and UV emissions during an X2.0 (left) and an M2.7 flare (right). (a1) and (a2) Show X-ray lightcurves from STIX and XRS. Excess Ly$\alpha $ emission measured by EUVS-B is shown in (b1) and (b2). Radiated energy for 304 Å against time for the combined ribbons and erupted material is plotted in (c1) and (c2). Divided ribbon and eruption radiated energy are plotted in (d1) and (d2). Dashed black lines in each panel represent the time of peak emission from each event eruption.

Figure 8 shows lightcurves for the X2.0 and M2.7 events, which both had eruption emission peak more than 5 minutes after the flare ribbons. For the X2.0 event, the peak of the 304 Å eruption emission occurs with no associated HXR emission as observed by STIX, providing a potential signature of eruption emission in Sun-as-a-star data. However, the eruption and ribbon contributions for this event cannot be delineated in photometric Ly$\alpha $ observations, as the increase in eruption emission does not show a sharp peak.

In contrast, during the M2.7 event, peak emission of erupted material in 304 Å occurred between peaks in ribbon emission. This later peak in ribbon emission does not correlate with a burst in HXRs, potentially suggesting this secondary peak was driven by thermal conduction of energy from the flare loops to the ribbons. Hence, comparison of photometric enhancements to HXR data may not allow eruption and ribbon contributions to be reliably distinguished during flares.

## Discussion and Conclusions

In this work, we provide an analysis of nine flares with associated bright eruptions. Three events were studied in detail, including an M2.0 event with an eruption seen on disk, an X9.0 event with a relatively less dynamic eruption, and an eruptive M1.1 event with its ribbons fully occulted from Earth’s viewpoint. Basic statistics on the radiative contributions of eruptions are provided from the full sample, using AIA data. Over the impulsive phase, the average percentage of total flare energy radiated by the eruptions was between $10\%$ in the hot 131 Å channel and $24\%$ in the relatively cooler 171 Å channel, with averages of $21\%$ and $13\%$ for the 304 Å and 1600 Å channels, respectively. This demonstrates that erupted material can radiate a substantial fraction of the total radiated energy in UV wavelengths during flares. However, it is noted that many of the flares in an initial sample of 82 events did not display a bright eruption, suggesting that this phenomenon is rare. As these eruptions can move at hundreds of $\mathrm{km}\,\mathrm{s}^{-1}$ (e.g. McCauley et al. 2015), they may drive blueshifts in Sun-as-a-star flare spectra, which could otherwise be interpreted as a signature of chromospheric evaporation (Batchelor and Hindsley 1991; Majury and Milligan 2025). This eruption-driven effect may also drive similar shifts in observations of stellar flares (Gunn et al. 1994; Berdyugina, Ilyin, and Tuominen 1999; Wang et al. 2024). Identification of eruptive events via coronal dimming may help clarify whether observed shifts are likely due to eruptions or evaporation in spatially integrated data (Veronig et al. 2021).

Synthesised flare profiles from contemporary flare simulations such as RADYN (Allred et al. 2005; Allred, Kowalski, and Carlsson 2015), HYDRO2GEN (Druett and Zharkova 2018, 2019), and FLARIX (Varady et al. 2010; Heinzel et al. 2016) may provide an avenue to disentangle ribbon and eruption flare contributions to observed flare irradiance and spectral variability, as these simulations typically do not include eruptions. Furthermore, the inclusion of eruptions in contemporary flare simulations may help clarify the physical mechanisms driving observed emissions. This may be possible through further development of existing MHD simulations of solar eruptive events (e.g. Ruan, Xia, and Keppens 2020; Jenkins and Keppens 2021, 2022; Jenkins, Osborne, and Keppens 2023; Druett, Ruan, and Keppens 2024).

While the eruptions in this sample were found to radiate a substantial fraction of the radiated energy, the total excess radiated energies were comparable to those seen in confined events (Greatorex, Milligan, and Chamberlin 2023). Thus, it remains unclear whether eruptive events tend to radiate a larger fraction of energy released during flares than events without eruptions. Comparative analysis of the energy release in eruptive and confined flares may further clarify whether eruptive events radiate released flare energy more efficiently.

For three of the nine events, HXR images were generated, providing insight into the heating mechanism driving enhanced emission from their associated eruptions. For the M2.0 event on 2 March 2022, we find only limited overlap of HXR emission and erupted material, despite the material radiating a large fraction of the excess radiated energy. Hence, it is unclear whether local nonthermal heating was primarily responsible for the enhancement of the erupted material. The X9.0 event showed no overlap of HXR sources with erupted material. This suggests the enhancement of the material was not strongly driven by nonthermal heating. It may be possible that there was substantial HXR emission from the erupted material that could not have been easily detected due to the dynamic range of STIX and HXI. However, we found no HXR emission during the occulted M1.1 event, further suggesting the heating mechanism for the observed bright eruptions could not have been nonthermal particle heating alone.

For the occulted M1.1 event, it seems unlikely that its eruption was heated through thermal conduction of energy from the flare ribbons, as the eruption peaked in brightness before the ribbons. Other heating mechanisms, such as Ohmic heating or heating by MHD waves, may have instead produced the observed brightening (Xue et al. 2016; Reeves et al. 2019). However, further observations and modelling are required to confirm this. The emission from the occulted M1.1 event somewhat resembles the bright eruption observed in Hayes et al. (2024), although, the event detailed in their study had thermal and nonthermal X-ray emission cospatial with erupted material, with the authors suggesting the heating mechanism to be deposition of energy by upwardly accelerated electrons with an inferred nonthermal energy of $\sim 2\times 10^{28}\,\mathrm{erg}$. The total energy radiated by the occulted M1.1 event eruption over the GOES period in 304 Å emission was $1.93\times 10^{28}\,\mathrm{erg}$, which is on the order of the average energy radiated by ribbons in the sample of $7.84\times 10^{28}\,\mathrm{erg}$, despite the event having the joint smallest GOES class in the sample. As He ii 304 Å emission strongly contributes to flare heating of the Earth’s ionosphere, similarly occulted events may produce unexpectedly strong ionospheric enhancements for their GOES class (Watanabe et al. 2021; O’Hare et al. 2025). If the flare loops are fully occulted but the bright erupted material is still visible, an ionospheric disturbance without a GOES classified flare may be observed. A search for such an event in AIA observations and subsequent analysis of total ionospheric electron content may further clarify the importance of this phenomenon to space weather.

Further insights into the radiative properties of eruptions may be provided via analysis of imaging spectrometry observations from existing instruments such as the EUV Imaging Spectrometer on Hinode (Hinode/EIS; Culhane et al. 2007) and the Interface Region Imaging Spectrograph (IRIS; De Pontieu et al. 2014), along with upcoming instruments such as the EUV High-throughput Spectroscopic Telescope on Solar-C (Solar-C/EUVST; Shimizu et al. 2019). Such studies may provide further insight into the contribution of eruption emission to observed line profile shifts in Sun-as-a-star observations, and guide the interpretation of observations of line shifts in stellar flares. Analysis of spectral observations may also help constrain the heating mechanism of erupted material through measurement of nonthermal broadening (Russell 2024).

In summary, this work finds that a substantial amount of flare excess radiated energy in UV emissions can originate from erupted material. We demonstrate that the brightening of erupted material is unlikely to be ubiquitously driven by nonthermal particle heating. Our work has implications for the interpretation of solar and stellar flare observations, with both eruptions and chromospheric evaporation potentially driving line shifts. Future observational and modelling work may provide further insights into the relative importance of these bright eruptions in observed spectral variability and elucidate the typical heating source responsible for the observed brightening.

## Data Availability

SolO/EUI data are available from the Solar Orbiter Archive (https://soar.esac.esa.int/soar/) and are also hosted on the Solar Influences Data Analysis Center website (https://sidc.be/EUI/data/releases/). EUVI data are available from the Stereo Science Centre (https://stereo-ssc.nascom.nasa.gov/data.shtml). The CACTus CME catalogue is accessible at https://www.sidc.be/cactus/. STIX data are available from the STIX data center (https://datacenter.stix.i4ds.net/). HXI data are available on request from the ASO-S mission website (http://aso-s.pmo.ac.cn/sodc/dataArchive.jsp). GOES/EUVS-B data are available on the NOAA archive (https://data.ngdc.noaa.gov/platforms/solar-space-observing-satellites/goes/). LYRA data are available at https://proba2.sidc.be/data/LYRA.
